# Incidence of sexually transmitted infections in men who have sex with men and who are at substantial risk of HIV infection – A meta-analysis of data from trials and observational studies of HIV pre-exposure prophylaxis

**DOI:** 10.1371/journal.pone.0208107

**Published:** 2018-12-03

**Authors:** Ricardo Niklas Werner, Matthew Gaskins, Alexander Nast, Corinna Dressler

**Affiliations:** Charité–Universitätsmedizin Berlin, corporate member of Freie Universität Berlin, Humboldt-Universität zu Berlin and Berlin Institute of Health; Department of Dermatology, Venerology and Allergy; Division of Evidence-Based Medicine (dEBM), Berlin, Germany; KEMRI Wellcome Trust Research Programme, KENYA

## Abstract

**Background:**

Men who have sex with men (MSM) and who engage in condomless anal intercourse with casual partners are at high risk of acquiring sexually transmitted infections (STIs), but reliable epidemiological data are scarce. Studies on HIV pre-exposure prophylaxis (PrEP) enrol MSM who indicate that they engage in behaviour that puts them at high risk of acquiring HIV. Because they also screen for STIs at regular intervals, these studies may serve as a valuable source to estimate incidence rates of STIs in this subpopulation of MSM.

**Methods:**

We systematically searched for trials and observational studies of PrEP in MSM that reported data on the incidence of STIs during the study period. Incidence rates were calculated as events per 100 person-years (py) with 95% confidence intervals (CI). Data from individual studies were pooled building subgroups along study types and geography. We performed sensitivity analyses, including data only from studies that met pre-defined quality criteria.

**Results:**

Twenty-four publications on 20 studies were included. The majority of studies reported that sexual behaviour and/or STI incidence remained stable or decreased during the study period. For syphilis, incidence rates ranged from 1.8/100py to 14.9/100py, the pooled estimate was 9.1/100py (95%-CI: 7.7–10.9). Incidence rates for gonorrhoea and chlamydia of any site ranged from 13.3/100py to 43.0/100py and 15.1/100py to 48.5/100py, respectively. Considering only studies that met the criteria for sensitivity analysis yielded pooled estimates of 39.6/100py (95%-CI: 32.9–47.6) and 41.8/100py (95%-CI: 33.9–51.5), respectively. The overall estimate for hepatitis C incidence was 1.3/100py (95%-CI: 1.0–1.8).

**Conclusions:**

Despite partly heterogeneous results, the data depict high incidence rates of STIs among MSM who engage in higher-risk sexual behaviours such as condomless sex with casual partners. This subpopulation of MSM requires access to STI screening at close intervals. By offering access to structures that provide regular STI monitoring and prompt treatment, PrEP may not only decrease HIV incidence but also have beneficial effects in decreasing the burden of STIs.

## Introduction

Data on the epidemiology of sexually transmitted infections (STIs) suggest a high burden of disease worldwide [1]. Increases in the incidence of chlamydia, gonorrhoea and syphilis infections have been observed in numerous countries, particularly among men who have sex with men (MSM) [2–6]. At the same time, the decreasing susceptibility of bacterial pathogens such as *Neisseria gonorrhoeae* [7–11] and *Mycoplasma genitalium* [12,13] to antimicrobials is becoming a major public health problem, especially given evidence that the risk of HIV transmission is higher in the presence of other STIs [14–17].

Reliable data on the incidence of STIs among sexual minorities are difficult to obtain [18–20]. In some countries, reporting systems for STIs may include information on the probable route of transmission. However, calculating incidence rates for STIs among MSM from these population-based data can rely only on reported cases for the numerator and estimates of the denominator, which is unknown (i.e., the total number of MSM at risk of acquiring an STI) [21,22]. Therefore, from an epidemiological viewpoint, a more reliable method to generate estimates of STI incidence rates in sexual minorities is to use incidence data from longitudinal cohort studies.

In the present systematic review and meta-analysis, we aimed to generate effect estimates for incidence rates of STIs in MSM who engage in sexual behaviours that put them at high risk of acquiring STIs. To do so, we used data on the incidence of STIs diagnosed during the study period in participants from studies on HIV pre-exposure prophylaxis (PrEP). PrEP is a biomedical form of primary HIV prevention that has been shown to be highly effective in clinical trials [23–26] and cohort studies [27–35]. Most PrEP trials and cohort studies only enrol MSM who indicate that they engage in behaviour that puts them at high risk of acquiring HIV, which is usually defined in these studies as reporting having had condomless anal intercourse within a specified amount of time with a specified minimum number of partners. Because they also screen for STIs at regular intervals, these studies may serve as a valuable source of STI incidence data in this subpopulation of MSM.

## Materials and methods

We conducted a systematic review and meta-analysis of data on the incidence of STIs in published trials and cohort studies of PrEP in MSM at risk of HIV infection. A review protocol was developed and followed but not published (S1 File).

### Eligibility criteria

Publications were eligible if they reported results from clinical studies of PrEP in MSM at risk for HIV infection and had a follow-up period of at least three months. To be included, studies had to report the incidence of STIs other than HIV and provide sufficient data on the person-years of follow-up to calculate incidence rates. Any form of testing for STIs had to be performed at regular intervals; studies that relied exclusively on self-reported incidence data were not included. We considered full-text publications and conference abstracts. Language was restricted to English, French and German.

### Information sources and search strategy

We searched Ovid MEDLINE, Embase and Cochrane CENTRAL on 28 September 2017 and conducted an update search on 25 July 2018 covering the period from the inception of the data base through the date of the search. Autoalerts for all searches were included through 1 August 2018. The full search strategies can be viewed in the online supplement (S1 File). Resource limitations meant that authors were contacted for missing data only for studies that had been included in the original search.

### Study selection and data collection

We checked all identified records for eligibility using a two-step process, screening titles and abstracts first, and assessing full texts of records included thereafter. Data were extracted by two evaluators (RNW and CD). Disagreement was resolved by discussion or by involving a third investigator (AN, MG). Apart from incident STI diagnoses and corresponding person-years of follow up, data were extracted on study type and characteristics, baseline characteristics of the sample, and any reported changes in sexual behaviour or STI incidence. We did not extract data that were only presented graphically. As data from a randomised controlled trial suggests that PrEP could have an effect on the incidence of genital herpes [36], we considered data on genital herpes only if these were reported separately for placebo or non-intervention arms. For data on hepatitis B, we chose the same approach since tenofovir is an established medication for chronic hepatitis B.

### Assumptions, simplifications and calculations

If incidence rates with 95% confidence intervals (95%-CIs) and the total person-years of follow-up were not reported, we calculated person-years as follows: If possible, we multiplied the number of participants per visit by the length of time since the previous visit, or by multiplying the number of participants by the mean follow-up time. Where only the overall number of participants at baseline and at the end of follow-up were reported and the number of participants for each time point was missing, we calculated person-years by multiplying the number of participants who completed the study plus half the number of participants who had not completed the closing visit by the length of the follow-up period. For all of these approaches, we used the number of incident diagnoses whenever possible; however, in some cases only the number of participants infected was available.

Incidence rates were calculated as events per 100 person-years of follow-up. Assuming a Poisson distribution, we used log incidence rates and corresponding standard errors to determine incidence rates with 95%-CIs. If no incident cases occurred during follow-up, we added a correction of 0.5 to the number of cases and person-years of follow-up as described in similar studies [37,38].

### Synthesis of the results

STI incidence data were grouped according to study type and geography. We calculated pooled estimates of the incidence rates and 95% CIs using a random effects model to account for methodological and clinical differences between the studies. Heterogeneity was quantified by means of the Higgins’s I-squared test statistic [39]. All calculations were performed using Stata SE 14 (metan command package).

### Assessment of the quality of the data

In order to evaluate the quality of the data, three criteria were established: First, we evaluated whether the data on incidence rates or numbers of incident STI diagnoses and person-years of follow-up were explicitly reported or easy to calculate or interpret. Second, we assessed whether studies reported having performed screening procedures for the detection of STIs during the follow-up. To satisfy this quality criterion, studies had to explicitly state having performed screening procedures at a minimum of six monthly intervals and in accordance with the Centers for Disease Control and Prevention (CDC) recommendations for STI screening in sexually active MSM [40]. Third, we evaluated the size of each study, considering more than 500 person-years of follow-up to be an indicator of higher quality incidence data.

### Further analyses

To examine sources of heterogeneity, we separately evaluated subgroups along study design and geography. We performed a sensitivity analysis for each outcome, only considering data extracted from studies that explicitly reported incidence rates (or provided easy-to-interpret data for numbers of incident diagnoses and person-years of follow-up) and that reported having applied STI screening methods that met our quality criterion #2. We did not undertake any formal tests of publication bias in our meta-analysis because our outcome of interest (i.e., STI incidence rates) was not an efficacy outcome that might have led to the selective publication of study results.

## Results

**Fig 1:**
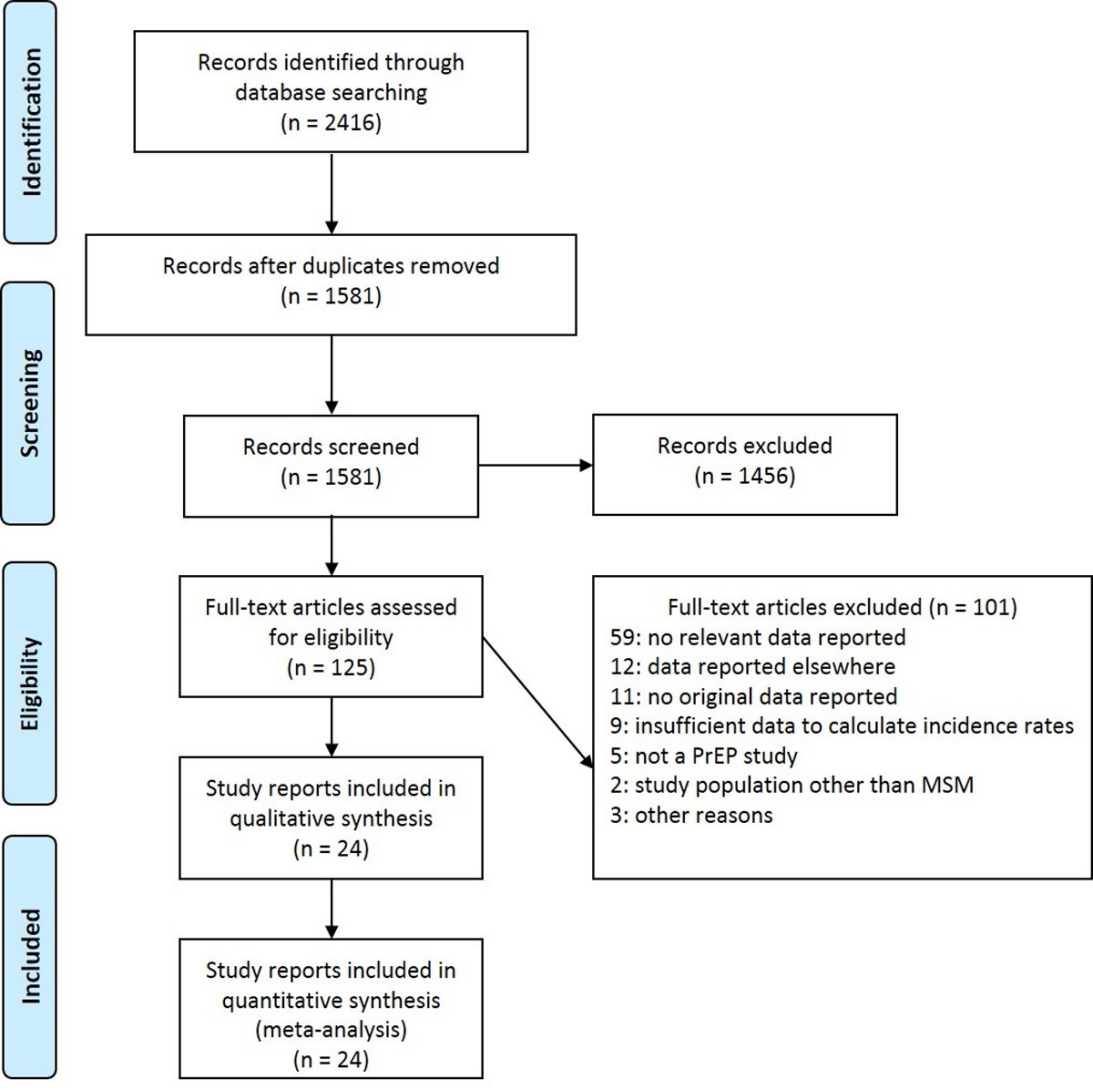
PRISMA flow diagram. Depicts the number of records identified, included and excluded, and the reasons for exclusions.

Our literature search yielded 2416 records, of which 1581 remained after duplicates had been removed. Of these, 1456 were excluded during the title and abstract screening, leaving 125 publications to be evaluated as full texts. Finally, 24 publications reporting the results of 20 studies were included. Fig 1 depicts the numbers of records identified, included and excluded [41].

### Study characteristics

The 20 studies were: three double-blind, placebo-controlled RCTs [23,24,26,42,43], one double-blind, active-controlled RCT [44], one open-label RCT [25] with STI incidence data derived from the PrEP group only, and 15 cohort studies [27–34,45–53]. A total of 11,918 participants were enrolled in these studies, yielding a cumulative 11,686.0 person-years of follow-up. Table 1 gives an overview of study types, inclusion criteria, sample sizes and baseline characteristics.

**Table 1:** Characteristics of the included studies.

Author, year (name of the study)	Population, geographic location, age in years	Type of study	Participants enrolled, follow-up	Safer sex counseling	Education / income	Alcohol and recreational drug consumption	Risk behaviour	STI at BL
**Double-blind, placebo-controlled RCTs; Participants not aware of group allocation**								
Grant et al. 2010 [23]; Solomon et al. 2014 [43]; Marcus et al. 2014 [42] (iPrEX)	MSM and transgender women at high risk for acquisition of HIV infection; Lima and Iquitos, Peru: 1400 (56.0%); Rio de Janeiro and Sao Paulo, Brazil: 370 (14.8%); Guayaquil, Ecuador: 300 (12.0%); San Francisco and Boston, USA: 227 (9.1%); Chiang Mai, Thailand: 114 (4.6%); Cape Town, South Africa: 88 (3.5%); mean age: 27.5 (verum), 26.8 (placebo); range: 18–67	Parallel-group RCT (daily TDF + FTC vs. placebo)	2499 randomized; 3324 person-years; duration of observation: median, 1.2 years	Risk-reduction counseling, free condoms, STI screening and treatment	- less than secondary: 523/2499; - secondary: 883/2499; - post-secondary: 1064/2499	No. of alcoholic drinks (on days when subject drank): - 0: 390/2499; - 1–4: 693/2499; - 5+: 1353/2499	No. of partners (past 12w): mean 18±43; unprotected receptive anal intercourse (past 12w): 1485/2499; unprotected anal intercourse (past 6m): 2001/2499; trans-actional sex (past 6m): 1027	- serum HSV-2: 888/2484 (35.75%); - urine leukocyte esterase positive: 45/2499 (1.80%); - current Hep B infection: 13/2499 (0.52%); - syphilis: 333/2499 (13.33%)
Hosek et al. 2013 [24] (PrEPare)	Young (18-22y) MSM with at least one episode of unprotected anal intercourse within the last 12 months; Chicago, USA; mean age: 19.97 +/- 1.3	partly double-blind parallel-group RCT (daily TDF + FTC vs. placebo vs. no pill)	68 pts. were enrolled, 58 randomized (study enrolment was discontinued after discussion of the iPrEx study results)	Many Men, Many Voices (3MV) prevention intervention: risk-reduction counseling, free condoms, STI screening and treatment	High school diploma 18/58 (31.03%); some college: 30/58 (51.72%); unemployed 32/58 (55.17%); received some form of public assistance in their lifetime: 78%	n.r.	Unprotected anal intercourse (past 30 days): 24/58 (41.38%); transactional sex: 10/58 (17.24%)	syphilis: 5/58 (8.6%); chlamydia: 2/58 (3.4%); genital herpes: 1/58 (1.7%); molluscum contagiosum 1/58 (1.7%)
Molina et al. 2015 [26] (IPERGAY)	MSM and transgender women with history of unprotected anal sex with at least two partners during the past 6 months; Paris, Lyon, Nice, Tourcoing and Nantes, France: 357 (89.2%); Montreal, Canada: 43 (10.8%); median age: 35 (IQR 29–43) in verum and 34 (29–42) in placebo group	double-blind parallel-group RCT (on-demand TDF + FTC vs. placebo)	414 pts. randomized, 400 followed; 431.3 person-years of follow-up; median follow-up of 9.3 months	Risk-reduction counselling (according to the RESPECT risk-reduction model), free condoms and gel, STI screening and treatment	Postsecondary education: 287/400	>5 Alcoholic drinks per day in past month: 91/400; Use of recreational drugs: 177/400	median number of partners in past 2 m: 8; Median no. of episodes of sexual intercourse in past 4w: 10	Any STI: 111/400 (27.8%)
**Double-blind, active-controlled RCT; Participants not aware of group allocation**								
Gulick et al. 2017 [44]	MSM or transgender women who had anal intercourse without a condom in the previous 90 days; Baltimore, Boston, Cleveland, Chapel Hill, Los Angeles, Newark, New York, Philadelphia, Pittsburg, San Francisco, Seattle, San Juan (Puerto Rico) and Washington DC, USA; median age 30, range 18–70	double-blind RCT (daily maraviroc vs. daily maraviroc + FTC vs. daily maraviroc + TDF vs. daily TDF + FTC)	406 pts. randomized, 343 (84%) completed	risk-reduction counseling, condom distribution, and HIV testing	less than high school: 3%; high or trade school: 17%; some college: 36%; finished college: 31%; advanced degree: 13% full-time employment: 52%; part-time: 23%; unemployed: 25%	n.r.	n.r.	31 (8%) had an STI; chlamydia in 15 (4%); gonorrhea in 5 (1%); and syphilis in 14 (3%)
**Open-label, placebo-controlled RCT; Participants aware of group allocation, for calculation of STI incidence only data from PrEP group used**								
McCormack et al. 2016 [25] (PROUD)	MSM who had anal intercourse without a condom in the previous 90 days; Birmingham, Brighton, London, Manchester, Sheffield, and York, UK; median age 35 (IQR 29–43)	open-label RCT (daily TDF + FTC vs. deferred PrEP)	523 contributed to HIV incidence analysis (both study groups); 465 person-years of follow-up; 243 person-years of follow-up in the immediate group	Risk reduction interventions were offered according to routine practice at the clinic	327/540 (61%) university graduates	231/525 (44%): one or more drugs associated with sexual disinhibition (γ-hydroxybuty-rate, 4-methylmeth-cathinone, or methamphetamine) (past 90 days)	21% of participants allocated to immediate PrEP reported receptive anal sex with ten or more partners without a condom at one year	331/517 (64%) STI (previous 12m); 172/517 (33%) rectal gonorrhoea or chlamydia (previous 12m); 184/510 (36%): at least one course of PEP (previous 12m)
**Cohort studies of PrEP users**								
Bristow et al. 2018 [45] (abstract only)	MSM and transgender women at risk for HIV; South California (four urban medical centers), USA	cohort study	394 participants; 238 PY of follow-up (for Chlamydia of the throat) to 485.1 PY (for urethral gonorrhea)	n.r.	n.r.	n.r.	n.r.	n.r.
Cotte et al. 2018 [46]	HIV- and HCV-negative MSM enrolled in a PrEP program; France; median age 37 years (IQR 30–45)	cohort study	930 HIV-negative MSM were enrolled for PrEP; follow-up was available for 916 of these, accounting for 972 PY of follow-up	n.r.	n.r.	n.r.	n.r.	17 participants HCV infected at BL; prevalence 1.8%; 14 cured, 3 active HCV infection
Golub et al. 2016 [47] (SPARK) (abstract only)	MSM and transgender women at risk for HIV acquisition; New York, USA	cohort study (RCT on behavioral interventions to increase adherence to PrEP)	280 began PrEP; 179.5 person-years of follow-up	not reported (data were derived from an RCT on behavioral interventions; assignment to groups not reported in the present conference abstract)	n.r.	n.r.	n.r.	any STI: 31/280 (11.1%) rectal STI: 25/280 (8.9%) urethral STI: 5/280 (1.8%) syphilis: 3/280 (1.1%)
Grant et al. 2014 [31] (iPrEX_OLE)	MSM and transgender women at high risk for acquisition of HIV infection (from various PrEP trials); Lima and Iquitos, Peru: 562 (45.9%); Chicago, San Francisco, Boston, and Atlanta, USA: 224 (18.3%); Rio de Janeiro and Sao Paulo, Brazil: 192 (15.7%); Guayaquil, Ecuador: 153 (12.5%); Chiang Mai, Thailand: 54 (4.4%); Cape Town, South Africa: 40 (3.3%); no data on mean / median age; 59% of pts were 25–39 years old	cohort study (open-label extension of previous PrEP RCTs, offering all pts. daily TDF + FTC)	1603 pts. were enrolled, among these 1128 started PreP at enrolment, 378 never started PreP and 97 started PreP after enrolment	Counselling targeted PrEP-adherence and -reporting; this involved counselling for sexual health	Less than secondary: 327/1590; Secondary: 547/1590; Post-secondary: 716 /1590	Alcohol use: <Once a month: 144/1603; 1–4 drinks on days when drinking: 508/1603; ≥5 drinks on days when drinking: 324/1603 Methamphetamine use: 31/1593 Cocaine use: 133/1539	519/1603 reported condomless receptive anal intercourse	Syphilis rapid plasma reagin positive: 253/1603; Herpes simplex virus-2: 791/1603; Gonorrhoea: 31/1587
Grinsztejn et al. 2018 [32] (PrEP Brasil)	MSM and transgender women who reported one or more sexual risk criteria in the previous 12 months (eg, condomless anal sex with two or more partners, two or more episodes of anal sex with an HIV-infected partner, or history of STI diagnosis); Rio de Janeiro and Sao Pauo, Brazil no data on mean age; 47.6% aged between 25 and 34 years	cohort study PrEP (TDF + FTC)	450 participants were enrolled, 375 were retained at 48 weeks	Brief risk reduction counselling and a short adherence support session	Length of schooling, years <12: 115/450 (25.6%); >/ = 12: 335/450 (74.4%)	Binge drinking: 241/375 (64.3%); Stimulant use: 77/375 (20.5%)	≥2 condomless anal sex partners in previous 12 months: 294/365 (80.5%); Anal sex with HIV-infected partners: 223/450 (49.6%); STI in the previous 12 months: 207/450 (46%)	Rectal chlamydia: 36/450 (8%); rectal gonorrhea: 22/450 (5%)
Hoornenborg et al. 2018 [48,49] (abstracts only) (AmPrEP)	MSM and transgender persons who have sex with men, who were at least 18 years old and had one or more risk factors for HIV infection in the past 6 months; Amsterdam, Netherlands	cohort study (daily or event-driven PrEP with TDF + FTC)	376 participants were included in the analysis; for these participants median follow-up was 1.76py	n.r.	n.r.	n.r.	n.r.	n.r.
Hosek et al. 2017 [34]	Young (18–22 years) MSM who reported HIV transmission risk behavior (eg, condomless anal intercourse, multiple sexual partners, or recent STI) in the last six months; Baltimore, Boston, Chicago, Denver, Detroit, Houston, Los Angeles, Memphis, Miami, New Orleans, Philadelphia, and Tampa, USA mean age 20.2 (SD 1.3)	cohort study (daily TDF + FTC)	200 pts. were enrolled, and 58 prematurely discontinued; overall study retention was 71%, including premature discontinuations and those who were lost to follow-up.	Prevention services at each visit included risk reduction counseling, condoms, and an Integrated Next Step Counseling session.	completed some college: 45.5%; currently unempoyed: 30.1%	n.r.	Average sex partners in previous month: 5; Condomless sex previous month: 80.8%; Condomless receptive anal intercourse with last partner: 58.0%; Any positive STI test: 22%; Ever exchanged sex for money: 28.6%	22% of pts. were diagnosed with an STI
Hosek et al. 2017 [33] (PrEPare)	Young (15–17 years) MSM who reported condomless anal intercourse with a partner of unknown or positive HIV serostatus, anal intercourse with at least three male partners, exchange sex, or sexually transmitted infection in the last six months; Boston, Chicago, Denver, Los Angeles, New Orleans, and Philadelphia, USA; mean age 16.5 (SD 0.73)	cohort study (daily TDF + FTC)	78 pts. were enrolled, 72 (92%) began daily oral PrEP and 46 (64%) of these completed 48 weeks of follow-up	Evidence-based personalized cognitive counselling intervention to reduce sexual risk and comprehensive HIV prevention package (including HIV testing, sexual health and adherence promotion using Integrated Next Step Counselling, free condoms, and safety assessments)	Currently attending school: 56/78 (72%); Eighth grade or less: 3/78 (4%); More than eighth grade but high school not completed: 58/78 (74%); GED: 3/78 (4%); High school diploma: 11/78 (14%); Some college: 1/78 (1%)	Alcohol consumption in the past month every week or more: 9/78 (12%); Got drunk in the past month once or more: 33/78 (67%); Smoked marijuana in the past month once or more: 47/78 (63%)	Have been paid for sex (lifetime): 13/78 (17%); unprotected receptive anal sex: 24 (60%); median number of male partners with sexual contacts: 1 (IQR: 1–2)	19 prevalent STIs were diagnosed in 14/78 pts (18%); rectal gonorrhea: 5/78 (6.4%); rectal chlamydia: 8/78 (10.3%); urethral chlamydia 4/78 (5.1%); syphilis 2/78 (2.6%)
Lal et al. 2017 [27] (VicPrEP)	MSM at risk of HIV infection (condomless receptive or insertive intercourse with an HIV seropositive person, receptive condomless anal intercourse with casual partners of unknown HIV status, or uncircumcised, condomless insertive anal intercourse with casual partners of unknown HIV status); Melbourne, Australia; median age 34.0 (IQR 30.8–45.0)	cohort study (combined daily TDF + FTC)	114 pts. were enrolled; STI data from 105 pts. were available at month 12; 107.2 person-years of follow-up	Safer sex practices, including condom use, were recommended at each study visit	64.1% had completed an undergraduate or higher degree	n.r.	97/114 had casual partners, with a mean of 19.2 anal sex acts in the past 3 months	12.3% had a new STI diagnosis at baseline
Lalley-Chareczko et al. 2018 [50]	Young (18–30 years) MSM and transgender women of colour; Philadelphia, USA; mean age 22.1 (range 18–29 years; SD 2.97)	cohort study	50 participants enrolled; 90% retention at 12 weeks, 74% retention at 24 weeks, 70% retention at 36 and 48 weeks	Standard HIV prevention services including condom provision, risk reduction counseling, HIV testing, and STI screening and treatment	n.r.	Drug/alcohol use: 37/50 (74%)	HIV-positive partner: 4 (8%); Partner(s) of unknown HIV status: 27 (54%); Inconsistent condom use: 40 (80%); History of STI: 29 (58%); Exchange of sex for commodities: 9 (18%); 4 or more partners in last 6 months: 15 (30%)	6 / 50 (12%) tested positive for rectal chlamydia and/or gonorrhea
Liu et al. 2016 [28] (US PrEP Demonstration Project)	MSM and transgender women who reported any of the following in the last 12 months: condomless anal sex with ≥2 male or transgender female partners; ≥2 episodes of anal sex with ≥1 HIV-infected partner; or sex with a male/transgender female partner and having a diagnosis of syphilis or rectal gonorrhea or chlamydia; San Francisco / Miami / Washington, USA; no data on mean / median age; 63% of pts were 26–45 years old	cohort study (daily TDF + FTC)	557 pts. enrolled, 437 pts. were retained in the study; 481 person-years of follow-up	client-centered risk-reduction counseling, free condoms and lubricants, linkages to appropriate community services	High school or less: 55/437; Some college: 119/437; College graduate: 156/437; Any postgraduate: 107/437; <20,000$: 128/437; 20,000–59 999$: 158/437; ≥60,000: 139/437	recreational drug use: 326/437; polysubstance use: 94/437; amphetamine use: 71/437; injected drug use: 7/437; alcohol (≥5 drinks per session, past 3m): 47/437	mean number of anal sex partners (past 3 months): 10.9; condomless receptive anal intercourse: 365/557 (65.5%)	147/557 (26.4%) had early syphilis, N. gonorrhoeae, or C. trachomatis at baseline
Marcus et al. 2016 [29] (‘Kaiser Cohort Northern California’) Comment below	PrEP users (Kaiser Permanente NC members); Northern California, USA; mean age 37.5 years (SD 10.1, range 18–68)	cohort study (daily TDF + FTC)	972 PrEP initiators; 850 person-years of PrEP use; mean duration during study period was 0.9 years per person	adherence support	% without high school diploma in census block, mean (SD) 11.5 (11.1); median household income in census block, USD (IQR) 74,094 (52,273–99,231)	history of alcohol/drug abuse: 6.3%	n.s.	STI at baseline: 15.9%
Molina et al. 2017 [30] (IPERGAY open-label extension)	MSM and transgender women with history of unprotected anal sex with at least two partners during the past 6 months; Paris, Lyon, Nice, Tourcoing, and Nantes France: 320 (89%); Montreal, Canada: 41 (11%); median age 37 years (IQR 30–44)	cohort study (on-demand TDF + FTC)	361 were enrolled; 63 (17%) prematurely discontinued; median follow-up time was 18.4 months (IQR 17.7–19.1); 518 person-years of follow-up for the assessment of HIV incidence	Comprehensive package of prevention services, including face-to-face risk-reduction counselling done by a peer community member, and free condoms and gel.	Postsecondary school: 324/355 (91%); Other: 31/355 (9%)	Use of recreational drugs for sex: 157/356 (44%)	No. of partners in past 2 months: median 7 (IQR 3–15); No of sexual acts in past 4 weeks: median 9.5 (IQR 5–15); Condomless receptive anal sex in most recent sex act: 136/176 (77%)	n.r.
Nguyen et al. 2018 [51]	PrEP users, considered at high risk based on reporting at least one seropositive sexual partner with a detectable viral load, or engaging in condomless anal sex with multiple partners whose HIV status was unknown. Montreal, Canada; median age 36 years (IQR 31–44)	Retrospective cohort study (PrEP, offered as a once-daily or intermittent regimen; evaluation of STI incidence for participants for whom a 12 months follow-up was available)	109 participants; 109 PY of follow-up	n.r.	Education: - Primary 1 (1.4%) - Secondary 9 (12.5%) - College 11 (15.3%) - University 51 (70.8%) Income: - <$10 000 3 (3.4%) - $10 001–20 000 12 (13.6%) - $20 001–35 000 7 (8.0%) - $35 001–55 000 17 (19.3%) - $55 001–75 000 21 (23.9%) - >$75 000 28 (31.8%)	n.r.	Number of sexual partners within 12 months prior to PrEP: - Stable, median (IQR) 1 (1–2) - Casual, median (IQR) 20 (10–40)	n.r.
Noret et al. 2018 [52]	MSM or transgender persons who reported having had condomless anal sex with at least 2 different partners over the last 6 months, and/or an STI over the last 12 months, and/or multiple courses of PEP within the prior 12 months, and/or use of chemsex. Paris, France; median age 36 years (IQR 26–42)	cohort study (on demand or daily TDF + FTC)	1049 participants were enrolled; 887 (84.5%) were still under follow-up at the end of study; 486 person-years of follow-up	Comprehensive package of prevention services including free condoms and gel and patient-centered counseling for risk reduction performed by a peer community member, to discuss the risks of HIV and other STIs.	Post-secondary education: 880 / 1043 (84.4%)	Use of chemsex in the last 4 weeks: 437 / 1027 (42.6%); Use of chemsex at last sexual intercourse: 279 / 1016 (27.5%)	Number of partners in past 3 month: median: 10 (IQR: 5–10); Number of condomless sexual intercourses acts (prior 4 weeks): median: 4 (IQR: 1–10); Participants reporting condomless sex at last intercourse: 557 / 1045 (53.3%); Participants reporting condomless receptive anal sex at last intercourse: 315 / 1031 (30.6%)	at least one bacterial STI at BL: 146 / 998 (14.6%); Chronic Hep C: 9 / 998 (0.9%); Chronic Hep B: 5 / 998 (0.5%)
Volk et al. 2015 [53] ('Kaiser Cohort San Francisco')	PrEP users (Kaiser Permanente SF members); San Francisco, USA	cohort study (daily TDF + FTC)	485 PrEP users; 304 person-years of PrEP use	n.r.	n.r.	n.r.	n.r.	n.r.

BL, baseline; FTC, emtricitabine; m, months; n.r., not reported / not assessed; No., number; PrEP, pre-exposure prophylaxis; PY, person-years; STI, sexually transmitted infection; TDF, tenofovir disoproxil fumarate; w, weeks; y, years; Comment: Data on incidence rates of STI in the study by Dr. J. Marcus were derived from data supplied in personal communication.

### Quality of data

Data on incidence rates for some or all of the reported STIs were directly reported or easy to interpret in 14 studies [25,27–31,34,42,43,45–48,51–53]. Eighteen studies explicitly reported the application of screening methods as recommended by the CDC for some or all of the STIs assessed [24–31,33,34,42–47,50,51]. Six studies had at least 500 person-years of follow-up, fulfilling our criterion for large study size [23,29–31,42,43,46,48,49]. Table 2 gives an overview of our assessment of data quality for each study.

**Table 2:** Evaluation of the quality criteria.

Author / Year (Name of the study)	STI incidence data directly reported or easy to interpret	Robust screening methods for detection of STI	Large study size (> 500 person-years)
Double-blind, placebo-controlled RCTs			
Grant et al. 2010 [23]; Solomon et al. 2014 [43]; Marcus et al. 2014 [42] (iPrEX)	+ (syphilis) + (HSV-2 seroincidence) - (all other data)	+ (syphilis) + (HSV-2) - (gonorrhoea, chlamydia)	+
Hosek et al. 2013 [24] (PrEPare)	-	+	-
Molina et al. 2015 [26] (IPERGAY)	- (syphilis) - (gonorrhoea, chlamydia) + (hepatitis C)	+ (syphilis) + (gonorrhoea, chlamydia) - (hepatitis C)	-
Double-blind, active-controlled RCT			
Gulick et al. 2017 [44]	-	+	-
Open-label, placebo-controlled RCT; STI incidence data derived from PrEP group only			
McCormack et al. 2016 [25] (PROUD)	+	+	-
Cohort studies of PrEP users			
Bristow et al. 2018 [45]	+	+	-
Cotte et al. 2018 [45]	+	+	+
Golub et al. 2016 [47] (SPARK)	+	+	-
Grant et al. 2014 [31] (iPrEx_OLE)	+	+	+
Grinsztejn et al. 2018 [32] (PrEP Brasil)	-	-	-
Hoornenborg et al. 2018 [49] (AmPrEP)	+ (hepatitis C) - (bacterial STI)	-	+
Hosek et al. 2017 [34]	+	+	-
Hosek et al. 2017 [33] (PrEPare)	-	- (syphilis); + (gonorrhoea, chlamydia)	-
Lal et al. 2017 [27] (VicPrEP)	+	+ (syphilis) - (gonorrhoea) + (chlamydia)	-
Lalley-Chareczko et al. 2018 [50]	-	+	-
Liu et al. 2016 [28] (US PrEP Demonstration Project)	+	+	-
Marcus et al. 2016 [29] ('Kaiser Cohort Northern California')	+	+	+
Molina et al. 2017 [30] (IPERGAY open-label extension)	+ (hepatitis C) - (syphilis, gonorrhoea, chlamydia)	+ (gonorrhoea, chlamydia, syphilis) - (hepatitis C)	+
Nguyen et al. 2018 [51]	+	+	-
Noret et al. 2018 [52]	+	-	-
Volk et al. 2015 [53] ('Kaiser Cohort San Francisco')	+	-	-

+, quality criterion met; -, quality criterion not met

### Changes in sexual behaviour and STI incidence during the study period

Two RCTs [24,26] and four cohort studies [28,32,33,50] reported that sexual behaviour had not changed with respect to condom use and/or the number of sexual partners. Two of these studies [28,50] and one further cohort study [49] additionally reported no change in the incidence of STIs. One retrospective cohort study [51] reported an increase in STI incidence compared to the time before PrEP initiation. However, this increase was not significant after adjusting for the number of screening visits. A decrease in the number of sexual partners and instances of condomless anal intercourse was observed in an RCT [23] and its open-label extension [31]. A smaller overall STI incidence in the second compared to the first half of the study was reported in one cohort [34]. In contrast, decreasing rates of condom use were reported in three cohort studies [30,47,52], and increasing incidence rates for STIs observed in two cohort studies [27,29]. Four studies did not report on changes in sexual behaviour or STI incidence during the follow-up [25,44–46].

### Any STI

One active-controlled RCT [44] and seven cohort studies [27–29,33,34,49,51] reported data on the incidence of any STI diagnosis, comprising a total of 2105.7 person-years (py) of follow-up. One of these studies specifically reported the incidence of any bacterial STI diagnosis (gonorrhoea, chlamydia or syphilis) [49]. Incidence rates ranged from 33.0/100py (95%-CI: 27.5–39.6) in the active-controlled RCT [44] to 99.8/100py (95%-CI: 82.6–120.6) in an Australian cohort study [27], yielding a pooled estimate of 72.4/100py (95%-CI: 58.8–89.1, I^2^ = 94.9%). The heterogeneity of the incidence rates remained high when only cohort studies conducted in North America [28,29,33,34,50,51] were considered [pooled estimate 64.4/100py (95%-CI: 48.4–85.6), I^2^ = 95.9%]. One Dutch [49] and the above-mentioned Australian cohort study [27] had comparable incidence rates [97.8/100py (95%-CI: 89.2–107.2) and 99.8/100py (95%-CI: 82.6–120.6), respectively]. Looking separately at the four cohort studies that fulfilled our quality criteria for the sensitivity analysis [28,29,34,51], our estimates ranged from 66.4/100py (95%-CI: 54.9–80.5) to 90.7/100py (95%-CI: 84.5–97.3), yielding an overall pooled estimate of 84.4/100py (95%-CI: 75.6–94.2, I^2^ = 68.3%).

### Syphilis

**Fig 2:**
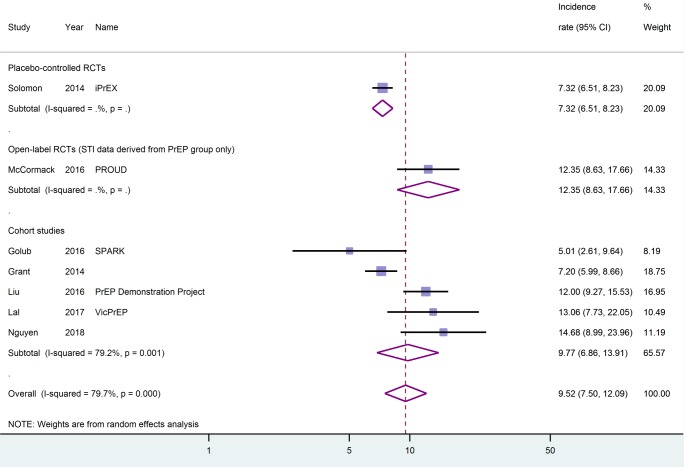
Syphilis incidence rates by study type, higher quality data only (sensitivity analysis). Incidence rates are given as events per 100 person-years. CI, confidence interval.

Three placebo-controlled RCTs [24,26,43], one active-controlled RCT [44], one open-label RCT [25], and eight cohort studies [27,28,31–33,47,50,51] reported on incident cases of syphilis, comprising a total of 7771.5 person-years of follow-up. Two studies reported on participants infected rather than number of infections, and re-infections may have been unreported in these studies [44,50]. Considering all studies reporting on the incidence of syphilis, we calculated a pooled estimate of 9.2/100py (95%-CI: 7.7–10.9, I^2^ = 65.7). Incidence rates ranged from 1.8/100py (95%-CI: 0.3–12.9) in a cohort study of 15-to-17-year-old PrEP users [33] to 14.9/100py (95%-CI: 5.6–39.8) in an RCT of PrEP in 18 to 22 year old participants [24]. As the person-years of follow-up were not directly reported in either of these two studies, these estimates should be treated with caution. Looking separately at cohort studies (i.e., in which all participants were on PrEP), the pooled estimate was 9.4/100py (95%-CI: 7.2–12.4, I^2^ = 68.2). Considering only the seven studies that met the quality criteria for our sensitivity analysis [25,27,28,31,43,47,51], we calculated a pooled estimate of 9.5/100py (95%-CI: 7.5–12.1, I^2^ = 79.7), see Fig 2. Looking separately at the estimates for different continents, the lowest pooled incidence rate was seen in studies that included participants from Latin America by majority [31,32,43] [7.4/100py (95%-CI: 6.7–8.2, I^2^ = 0.0)], and the highest incidence was seen in an Australian cohort study [27] [13.1/100py (95%-CI: 7.7–22.1)].

### Gonorrhoea and chlamydia

Data on the incidence of gonorrhoea and chlamydia were reported in three placebo-controlled RCTs [23,24,26] one active-controlled RCT [44] one open-label RCT [25] and in eight cohort studies [27–29,33,45,47,50,51]. Grant et al. 2010 reported only testing for urethral gonorrhoea and chlamydia infection upon positive urine leukocyte esterase [23] and Lal et al. reported testing for genital gonorrhoea only upon clinical suspicion [27]. For two studies, routine sampling did not include the throat [44] or it remained unclear, whether this site was included [45]. In one cohort study, screening tests only included anal and pharyngeal sites for gonorrhoea, and anal and urethral sites for chlamydia [27]. Three studies reported on persons infected, not on events of infection during follow-up [26,44,50]. In the following we have put the different anatomical locations in boldface to improve readability.

One cohort study reported data on the **combined outcome of urethral gonorrhoea and/or chlamydia** [47]. In 179.5 person-years of follow-up, 32 cases of urethral STIs were diagnosed, leading to an incidence rate of 17.8/100py (95%-CI: 12.6–25.2).

Two placebo-controlled RCTs [23,24] and four cohort studies [29,33,45,51] reported data on **urethral gonorrhoea**, with an overall follow-up of 3626.4 person-years. The incidence of urethral gonorrhoea ranged from 1.3/100py (95%-CI: 0.9–1.8) in the RCT that only tested for urethral gonorrhoea upon positive leukocyte esterase[23] to 6.4/100py (95%-CI: 3.1–13.5) in a retrospective Canadian cohort study.[51] The pooled estimate was 3.3/100py (95%-CI: 1.6–7.0, I^2^ = 88.4%). Looking separately at cohort studies, the pooled estimate was 4.3/100py (95%-CI: 2.3–8.0, I^2^ = 72.1%). Considering only the three cohort studies that met the quality criteria for inclusion in the sensitivity analysis [29,45,51], the pooled estimate was 4.0/100py (95%-CI: 1.8–8.8, I^2^ = 81.3%).

For the outcome of **urethral chlamydia**, additionally to the six above-mentioned studies reporting on urethral gonorrhoea, one cohort study [27] reported data, resulting in a total of 3706.0 person-years of follow-up. Incidence rates ranged from 1.0/100py (95%-CI: 0.7–1.5) to 26.2/100py (95%-CI: 12.5–54.9), and the pooled estimate was 7.5/100py (95%-CI: 3.4–16.3, I^2^ = 94.9%). Among the cohort studies [27,29,33,45,51], heterogeneity was lower, yielding a pooled estimate of 9.2/100py (95%-CI: 7.2–11.7, I^2^ = 31.6%). Considering the four cohort studies that met the criteria for inclusion in the sensitivity analysis [27,29,45,51], the overall estimate for urethral chlamydia infection was 9.1/100py (95%-CI: 6.8–12.2, I^2^ = 48.7%).

Four studies reported on the **combined incidence of rectal gonorrhoea and/or chlamydia** [25,27,47,50], with an overall follow-up of 564.8 person-years. Incidence rates from these reports ranged from 38.3/100py (95%-CI: 31.2–46.9) in the PrEP group of an English open-label RCT [25] to 70.0/100py (95%-CI: 55.8–87.7) in an Australian cohort study [27]. The two PrEP cohorts from the US [47,50] showed similar incidence rates [pooled estimate 51.9/100py (95%-CI: 43.1–62.5, I^2^ = 0)]. The pooled estimate from all studies was 52.6/100py (95%-CI: 39.6–69.7, I^2^ = 80.9%). Only considering the three studies that met the criteria for inclusion in the sensitivity analysis [25,27,47], the estimate for the incidence of rectal gonorrhoea and/or chlamydia was 51.1/100py (95%-CI: 36.5–71.5, I^2^ = 86.8%).

Five cohort studies reported the incidence of rectal gonorrhoea and rectal chlamydia separately [27,29,33,45,51]. For the outcome of rectal gonorrhoea, 1136.1 person-years of follow-up were available; for rectal chlamydia 1072.4 person-years. Incidence rates for **rectal gonorrhoea** ranged from 9.3/100py (95%-CI: 6.8–12.7) to 29.9/100py (95%-CI: 21.1–42.2), yielding a pooled estimate of 16.3/100py (95%-CI: 10.4–25.6, I^2^ = 87.2%). Considering the four studies [27,29,45,51] for sensitivity analysis only, the pooled estimate was 17.4/100py (95%-CI: 10.6–28.5, I^2^ = 89.8%). Incidence rates for **rectal chlamydia** ranged from 14.6/100py (95%-CI: 7.3–29.1) to 42.4/100py (95%-CI: 36.7–49.0). The pooled estimate was 30.3/100py (95%-CI: 21.9–42.0, I^2^ = 86.2%). Considering the four studies [27,29,45,51] for sensitivity analysis only, the pooled estimate for rectal chlamydia was 33.4/100py (95%-CI: 24.3–46.1, I^2^ = 86.8%). For both rectal gonorrhoea and rectal chlamydia, the separate analysis of geographic regions did not show less heterogeneous results.

**Fig 3:**
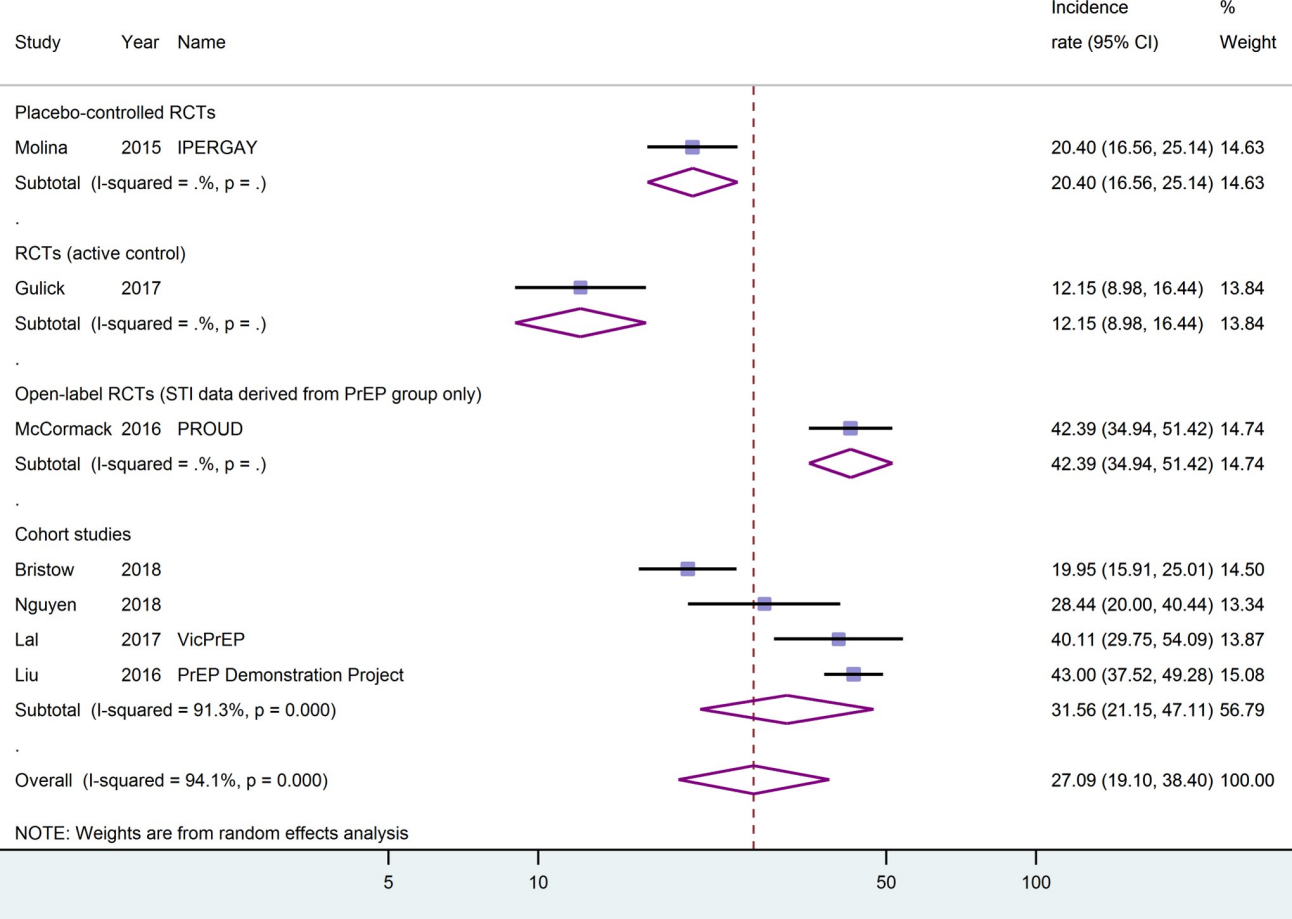
Gonorrhoea of any anatomical localisation by study type. Incidence rates are given as events per 100 person-years. CI, confidence interval.

One placebo-controlled RCT [26], one head-to-head RCT [44], one open-label RCT with data only derived from the PrEP group [25], and four cohort studies [27,28,45,51] reported on gonorrhoea and chlamydia of any of the body sites tested. The cumulative follow-up was 2093.2 and 2057.2 person-years, respectively. The incidence of **gonorrhoea of any site** ranged from 12.2/100py (95%-CI: 9.0–16.4) in an active-controlled RCT reporting the number of persons infected during follow-up rather than reporting the number of events of infection [44] to 43.0/100py (95%-CI: 37.5–49.3) in the US PrEP Demonstration Project [28]. The pooled estimate from all studies reporting this outcome was 27.1/100py (95%-CI: 19.1–38.4, I^2^ = 94.1%), see Fig 3. Looking separately at cohort studies, the pooled estimate was 31.6/100py (95%-CI: 21.2–47.1, I^2^ = 91.3%). The heterogeneity of the incidence rates remained high when different geographic regions were pooled separately. Three studies [25,28,51] had incidence data easily accessible and reported applying proper screening procedures for these outcomes (nucleic acid amplification tests performed on anal and throat swabs and urine samples). Considering these studies only, the pooled estimate was 39.6/100py (95%-CI: 32.9–47.6, I^2^ = 57.6%).

**Fig 4:**
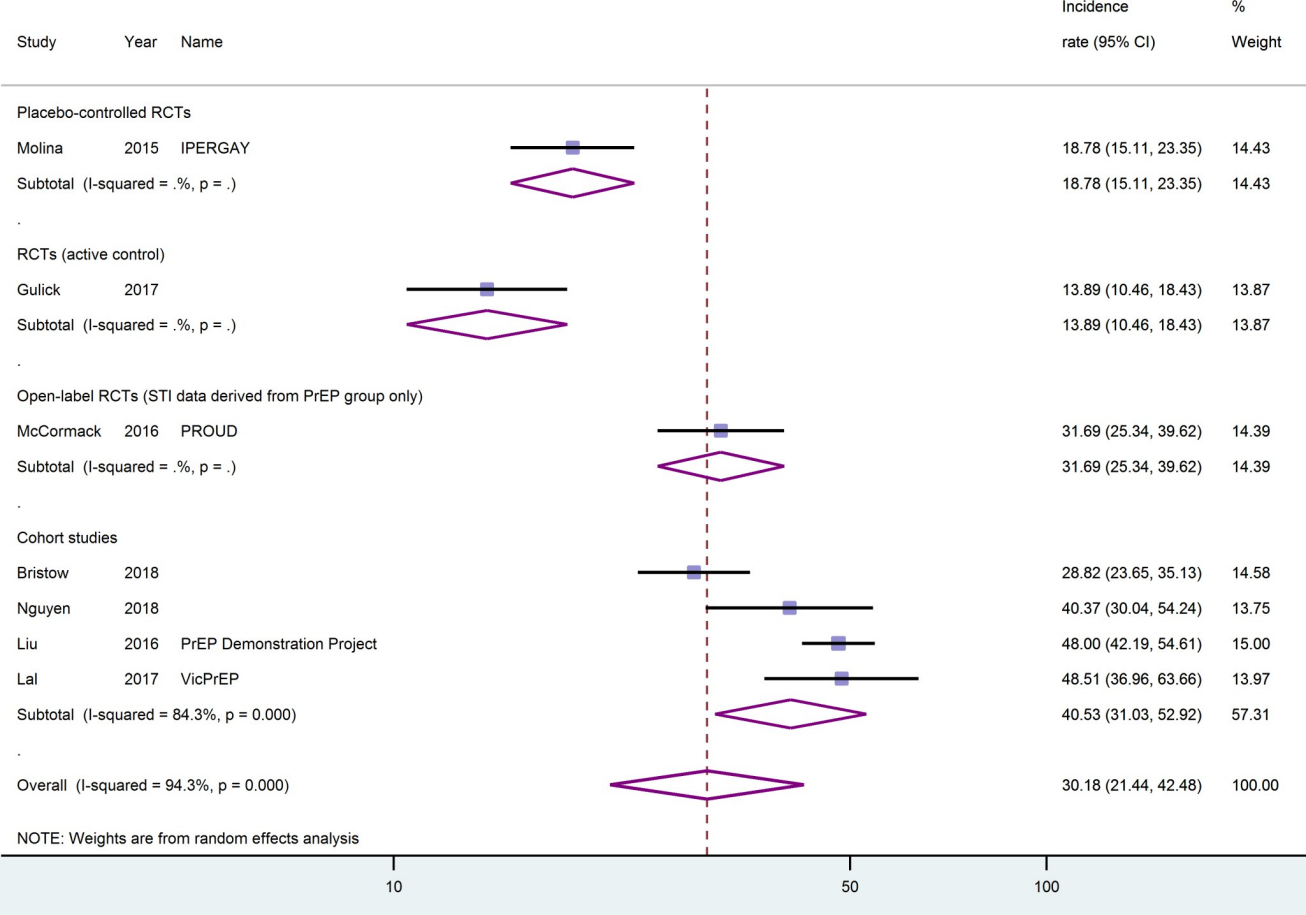
Chlamydia infections of any anatomical localisation by study type. Incidence rates are given as events per 100 person-years. CI, confidence interval.

For **chlamydia of any site**, incidence rates were similarly heterogeneous, ranging from 13.9/100py (95%-CI: 10.5–18.4) in the active-controlled RCT [44] to 48.5/100py (95%-CI: 37.0–63.7) in the Australian VicPrEP cohort [27]. The pooled estimate was 30.2/100py (95%-CI: 21.4–42.5, I^2^ = 94.3%), see Fig 4. Looking separately at cohort studies, the pooled estimate was 40.5/100py (95%-CI: 31.0–52.9, I^2^ = 84.3%). Only considering studies that met the criteria for the sensitivity analysis, the pooled estimate was 41.8/100py (95%-CI: 33.9–51.5, I^2^ = 72.3%).

### Mycoplasma and ureaplasma

No study reporting on the incidence of genital or rectal mycoplasma or ureaplasma infection was identified.

### Viral hepatitis

**Fig 5:**
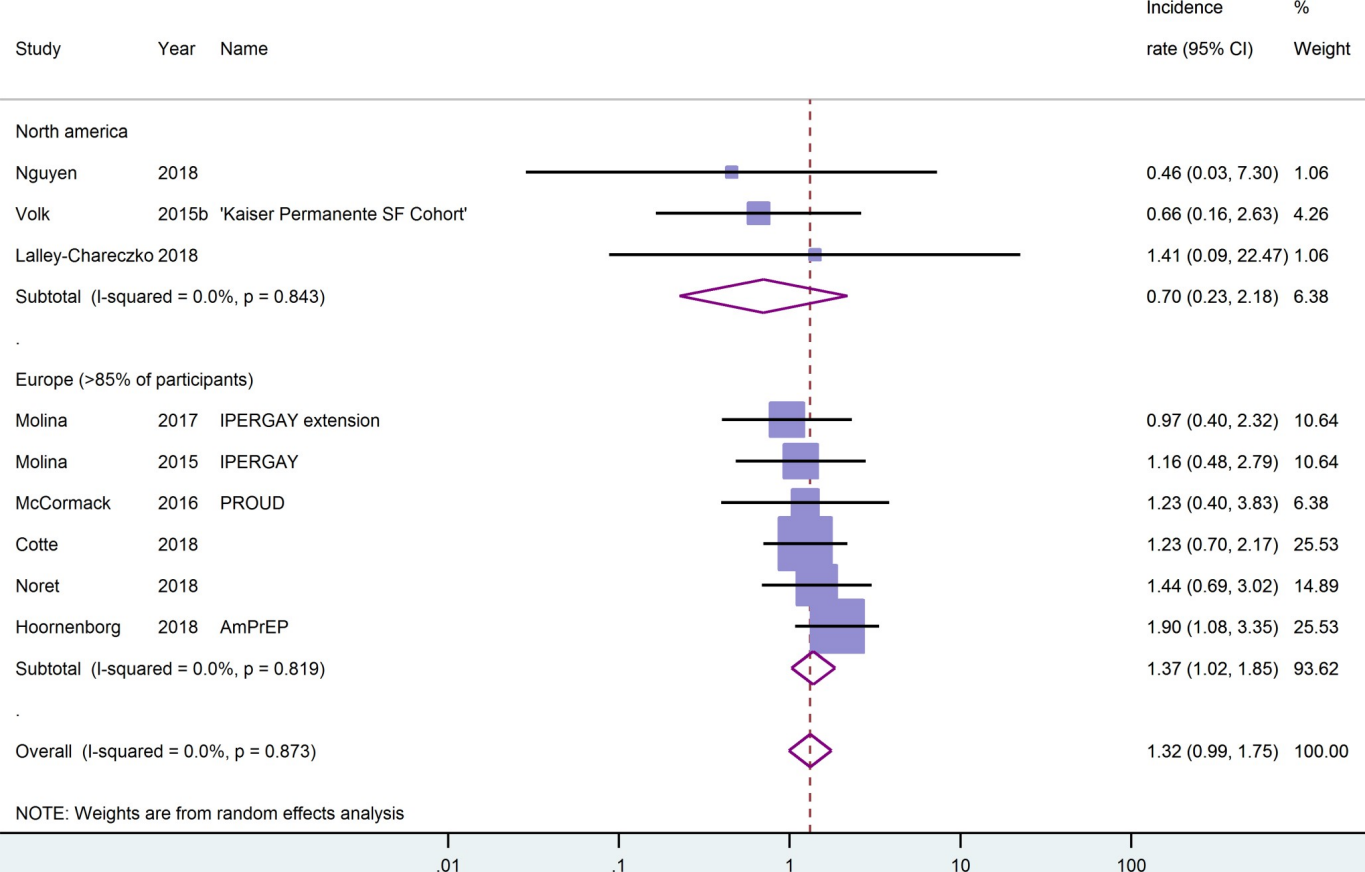
Hepatitis C incidence rates by geography. Incidence rates are given as events per 100 person-years. CI, confidence interval.

One placebo-controlled RCT [26], one open-label RCT [25], and seven cohort studies [30,46,48,50–53] reported data on the incidence of hepatitis C (HCV) during a cumulative follow-up of 3730.5 person-years. Of these studies, only five [25,30,46,51,52] reported using serology assays to screen for HCV at regular interviews. In the study by McCormack et al. [25] this applied only to participants who reported injecting or snorting drugs, fisting or the use of sex toys. The lowest HCV incidence was reported in a Canadian retrospective cohort study with zero events during 109 person-years of follow-up [51]. The highest HCV incidence rate was reported for the Dutch AmPrEP cohort [48] and totalled 1.9/100py (95%-CI: 1.1–3.4). The overall estimate for HCV incidence was 1.3/100py (95%-CI: 1.0–1.8, I^2^ = 0.0). Looking separately at the cohort studies, the pooled estimate was almost identical. Although the incidence rate of HCV in European studies was twice the incidence rate in North American studies, the confidence intervals for these estimates overlapped, see Fig 5. Pooling data only from the sensitivity analysis sample [25,30,46] yielded a pooled estimate for HCV incidence of 1.2/100py (95%-CI: 0.8–1.8, I^2^ = 0.0).

One RCT [26] reported no incident cases of **hepatitis B** during 175 person-years of follow-up in the placebo group.

The incidence of **hepatitis A** was reported in one French cohort study [52]. Six cases were diagnosed during a follow-up of 486 person-years, yielding an incidence rate of 1.2/100py (95%-CI: 0.6–2.8).

### Participants with at least one STI and other STIs reported

Six studies reported on the outcome ‘participants diagnosed with at least one STI’, and one study each reported on anogenital warts, herpes simplex seroincidence, molluscum contagiosum and trichomonas. These data are reported in the online supplement (S2 File).

## Discussion

The present meta-analysis aimed to generate estimates for incidence rates for various STIs among MSM who engage in high-risk sexual behaviours, such as condomless anal intercourse with casual partners. The results indicate that the incidence rates for STIs in this subgroup of MSM are high. The incidence rate for a diagnosis with any STI was about 84/100py in the four cohort studies that met our criteria for higher-quality data. The bulk of diagnoses with any STI were due to chlamydia and gonorrhoea infections at different anatomical sites. Here, rectal gonorrhoea, chlamydia or both played a major role, with a pooled incidence rate of more than 50/100py. This is worrying given the rising levels of antimicrobial resistance seen especially in Neisseria gonorrhoea in recent years [7–11]. Although Mycoplasma genitalium infections have recently received increased attention as a difficult-to-treat STI [12] and epidemiological research partly supports screening for this infection in populations at higher risk, such as MSM [12,54–56], no corresponding incidence data were reported in the studies included in our meta-analysis.

Our overall estimate for the incidence rate of hepatitis C infections was 1.3/100py. This is substantially higher than the incidence of 0.4 per 1000 persons per year seen in HIV-uninfected MSM and even the incidence of 7.8 per 1000 persons per year seen in HIV-positive MSM reported in a recent meta-analysis that included both prospective and retrospective studies [57]. Recent research suggests that only a proportion of the hepatitis C infections in this susceptible population are sexually transmitted and that practices involving intravenous or nasal drug use play an important role [58,59]. Due to the potentially severe sequelae and the high cost of treatment, our findings are worrying and suggest that policy makers may wish to consider redoubling efforts at primary prevention in those at highest risk (e.g. routine screening, needle exchange programmes).

In the one study reporting incidence data for hepatitis A, a rate of 1.2/100py was observed. While this is a high rate by any measure, it must be kept in mind that this French cohort study was conducted during an outbreak of hepatitis A among MSM in several European countries [60–65], including France [66]. Nevertheless, these data underline the importance of increasing hepatitis A vaccination coverage in MSM, specifically in those who engage in sexual behaviour that puts them at high risk of acquiring HIV and other STIs.

Our study does not attempt to compare STI incidence rates among MSM taking PrEP versus MSM not taking PrEP. One such comparison has been performed previously [67]. However, the validity of such studies is questionable [68]. To be eligible to participate in clinical trials of PrEP, participants had to be at high risk of infection with HIV (and hence other STIs) already. Comparing this group of MSM to other MSM who are not at high risk, or to the overall population of MSM, will lead to predictable and meaningless results. Moreover, the frequent testing for STIs in the PrEP studies will lead to an increase in the number of detected STI diagnoses. Indeed, one of the PrEP cohort studies included reported that screening every six months instead of quarterly would have delayed diagnosis of STIs in 24% of the participants [47]. A retrospective cohort study that compared the incidence of STIs in the year before PrEP initiation to the year after, found a significant increase only when the data were not adjusted for testing frequency [51]. Two longitudinal studies comparing the incidence of STIs before PrEP initiation to that during PrEP intake have reported mixed results: in the first, which assessed the incidence of rectal STIs, no increase was seen [69], whereas in the second, a modest but significant increase in ‘any STI’ and Chlamydia infections was reported [70]. Generally, the majority of studies included in our meta-analysis reported either a stable or decreased risk-behaviour and/or STI incidence during follow-up. This finding is supported by a recent case-crossover study [71].

Since the participants included in the studies benefit directly from their participation (free PrEP medication and STI testing), the follow-up may be more complete than in studies designed to assess STI incidence rates in susceptible sexual minorities. However, using data from PrEP trials and cohort studies to estimate STI incidence rates has a number of important limitations. First, the majority of studies included in our meta-analysis were not designed specifically to assess STI incidence rates. We have attempted to address this limitation with our sensitivity analysis. The results of this analysis generally support the validity of our approach. A second limitation is our inclusion of data from heterogeneous study designs, as well as different regions and populations of MSM. While participants included in double-blind placebo-controlled trials will not have known their group assignment, participants in the other types of studies, particularly in PrEP cohort studies, will all have been aware that they were using PrEP. This may have affected participants’ sexual risk taking behaviour and therefore their risk of acquiring STIs. We have accounted for this by building subgroups according to each of the different study designs and presenting the data separately for these where appropriate. Nevertheless, when interpreting the overall effect estimates, readers will need to bear this limitation in mind. The methodological and clinical heterogeneity is reflected by I-squared as a statistical tests for heterogeneity. This was often high, with values exceeding 75%. In a few cases, heterogeneity was substantially reduced when we analysed subgroups separately according to study design or region. While being at risk of acquiring HIV and therefore being eligible for PrEP was the common inclusion criterion for most of the studies, the actual risk behaviour of the participants may have varied between studies (and indeed over time) and may partly explain the heterogeneity of our results. Because the risk behaviour of participants in the included studies was not reported in ways that were consistent across the studies, comparing these risk behaviours is difficult and stratifying them in meaningful ways not feasible. Further factors, such as age and ethnicity of participants, may also partly explain high values of I-squared obtained for some of our pooled estimates.

If the limitations of our study are borne in mind, our estimates of STI incidence rates among MSM who are at high risk of STI infection can be helpful to policy makers and health professionals working in the fields of primary prevention and clinical care. For example, our estimates could be used to make recommendations on the frequency of STI screening and access to it in this susceptible population. Indeed, offering PrEP to this group of MSM may not only be a promising strategy to reduce the incidence of HIV. It could also help embed the target population into a comprehensive programme of frequent STI screening and treatment, potentially reducing the incidence and prevalence of STIs in the medium term, as predicted by a recent modelling study [72].

## Conclusions

Despite the heterogeneous designs of the studies included in our meta-analysis and the heterogeneity of some of our results, our overall findings suggest that the incidence rates of various STIs among MSM who engage in high-risk sexual behaviour is high. The high rates of gonorrhoea, chlamydia and hepatitis C infections in the included cohort studies raise particular concerns. This subgroup of MSM can benefit from access to STI testing and treatment at close intervals. The use of PrEP is a highly effective means of preventing HIV infection and should be embedded in a comprehensive programme targeting primary and secondary prevention and treatment of other STIs.

## Supporting information

S1 File Review protocol. (PDF)

S2 File Online supplement. Results on the outcomes ‘Participants diagnosed with at least one STI’, anogenital warts, herpes simplex and other STIs.
(PDF)

S3 File Data extracted from studies. (XLSX)

S4 File PRISMA checklist. (PDF)
