# Correction: Incidence of sexually transmitted infections in men who have sex with men and who are at substantial risk of HIV infection – A meta-analysis of data from trials and observational studies of HIV pre-exposure prophylaxis

**DOI:** 10.1371/journal.pone.0226209

**Published:** 2019-12-03

**Authors:** Ricardo Niklas Werner, Matthew Gaskins, Alexander Nast, Corinna Dressler

There are errors in the analysis of this study which affect some of the results but not the overall conclusions of the work. This study calculates estimates of the incidence of a range of sexually transmitted infections (STIs) among MSM who were at substantial risk of HIV infection by pooling data from all studies identified in the authors’ systematic review that met pre-defined inclusion criteria. The authors conducted sensitivity analyses that pooled data only from those studies that met the following pre-defined quality criteria:

1. Did the study report data on incidence rates or numbers of incident STI diagnoses and person-years of follow-up explicitly or in a way that was easy to calculate or interpret?
2. Did the study explicitly state that screening procedures had been undertaken at a minimum of six monthly intervals and using the tests / procedures recommended by the Centers for Disease Control and Prevention (CDC) for STI screening in sexually active MSM? (https://www.cdc.gov/std/tg2015/tg-2015-print.pdf)

Two studies were mistakenly classified as having fulfilled quality criterion no. 2 above: Molina et al. (2017) [ref. 30] and Noret at al. (2018) [ref. 52]. One study was mistakenly not classified as having fulfilled quality criterion no. 2: Hoornenborg et al. (2018) [ref. 48]. These mistakes resulted in errors in the estimation of the pooled incidence for hepatitis C in sensitivity analyses (Results section) as well as the study information provided in Table 2.

**Table 2:** Evaluation of the quality criteria.

Author / Year (Name of the study)	STI incidence data directly reported or easy to interpret	Robust screening methods for detection of STI	Large study size (> 500 person-years)
Double-blind, placebo-controlled RCTs			
Grant et al. 2010 [23]; Solomon et al. 2014 [43]; Marcus et al. 2014 [42] (iPrEX)	+ (syphilis) + (HSV-2 seroincidence) - (all other data)	+ (syphilis) + (HSV-2) - (gonorrhoea, chlamydia)	+
Hosek et al. 2013 [24] (PrEPare)	-	+ (urethral gonorrhoea, chlamydia) - (syphilis)	-
Molina et al. 2015 [26] (IPERGAY)	-	+ (syphilis, gonorrhoea, chlamydia) - (hepatitis C)	-
Double-blind, active-controlled RCT			
Gulick et al. 2017 [44]	-	+	-
Open-label, placebo-controlled RCT; STI incidence data derived from PrEP group only			
McCormack et al. 2016 [25] (PROUD)	+	+	-
Cohort studies of PrEP users			
Bristow et al. 2018 [45]	+	+	-
Cotte et al. 2018 [46]	+	+	+
Golub et al. 2016 [47] (SPARK)	+	+	-
Grant et al. 2014 [31] (iPrEx_OLE)	+	+	+
Grinsztejn et al. 2018 [32] (PrEP Brasil)	-	-	-
Hoornenborg et al. 2018 [48,49] (AmPrEP)	+	+ (hepatitis C) - (bacterial STI)	+
Hosek et al. 2017 [34]	+	- (syphilis); + (gonorrhoea, chlamydia)	-
Hosek et al. 2017 [33] (PrEPare)	-	- (syphilis); + (gonorrhoea, chlamydia)	-
Lal et al. 2017 [27] (VicPrEP)	+	+ (syphilis) - (gonorrhoea) + (chlamydia)	-
Lalley-Chareczko et al. 2018 [50]	-	+ (syphilis, gonorrhoea, chlamydia) - (hepatitis C)	-
Liu et al. 2016 [28] (US PrEP Demonstration Project)	+	+	-
Marcus et al. 2016 [29] ('Kaiser Cohort Northern California')	+	+	+
Molina et al. 2017 [30] (IPERGAY open-label extension)	- (syphilis, gonorrhoea, chlamydia) + (hepatitis C)	+ (syphilis, gonorrhoea, chlamydia) - (hepatitis C)	+
Nguyen et al. 2018 [51]	+	+	-
Noret et al. 2018 [52]	+	-	-
Volk et al. 2015 [53] ('Kaiser Cohort San Francisco')	+	-	-

+, quality criterion met; -, quality criterion not met

In the Viral hepatitis subsection of the Results, there are errors in the second and ninth sentences. The correct second sentence is: Of these studies, only four [25,46,48,51] reported using serology assays to screen for HCV at regular intervals. The correct ninth sentence is: Pooling data only from the sensitivity analysis sample [25,46,48,51] yielded a pooled estimate for HCV incidence of 1.5/100py (95%-CI: 1.0–2.1, I^2^ = 0.0).

In the Quality of data subsection of the Results, there are errors in the first two sentences. The correct first two sentences are: Data on incidence rates for some or all of the reported STIs were directly reported or easy to interpret in 15 studies [25,17–31,34,42,43,45–49,51–53]. Eighteen studies explicitly reported the application of screening methods as recommended by the CDC for some or all of the STIs assessed [24–31,33,34,42–48,50,51].

The authors clarified that they did not undertake any formal tests of publication bias in their meta-analysis because their outcome of interest (i.e., STI incidence rates) was not an efficacy outcome that might have led to the selective publication of studies. However, they state it is possible that the incidence rates of certain STIs in the published studies might have been so low that they were regarded as unimportant and therefore not reported, leading to reporting bias that might affect the meta-analysis. The authors acknowledge this as a potential limitation of their study.

There are a number of errors in Table 2. Please see the correct Table 2 below.
