# Supplementary material for: Incidence of sexually transmitted infections in men who have sex with men and who are at substantial risk of HIV infection – A meta-analysis of data from trials and observational studies of HIV pre-exposure prophylaxis
Source: PLoS One. 2018 Dec 3;13(12):e0208107. doi: 10.1371/journal.pone.0208107 (PMC6277101; doi:10.1371/journal.pone.0208107)
Supplement: S1 File — (PDF) [file pone.0208107.s001.pdf]

Titel: Incidence rates of sexually transmitted infections in men who have sex with men and who engage in ‘risky’ sexual behaviours – a meta-analysis of data published in trials and observational studies of HIV pre-exposure prophylaxis

Authors: Ricardo N. Werner[1], Matthew Gaskins[1], Alexander Nast[1], Corinna Dressler[1]

[1] Charité – Universitätsmedizin Berlin, corporate member of Freie Universität Berlin, Humboldt-Universität zu Berlin, and Berlin Institute of Health, Department of Dermatology, Venerology und Allergology, Division of Evidence based Medicine (dEBM)

S1 File: Review protocol

1. Rationale and review question 2
2. Search for studies 2
2.1. Databases and search dates 2
2.2. Search strategies 2
3. Eligibility criteria for the inclusion of studies 4
4. Methods for study selection and data collection 4
5. Assessment of the quality of the data 5
6. Calculation of incidence rates from studies 5
7. Synthesis of the results from each study 6
8. Heterogeneity and sensitivity analyses 6
9. References 7

1. Rationale and review question

We will conduct a systematic review and meta-analysis of data on the incidence of sexually transmittable infections (STIs) in published trials and cohort studies of PrEP in men who have sex with men (MSM) at risk of HIV infection.

Reliable epidemiologic data on incidence rates of STIs among MSM and particularly among MSM who engage in condomless anal sex are scarce. It remains a challenge to obtain representative and unbiased samples of sexual minorities[1-3] and using overall epidemiologic country-specific STI incidence data to calculate population-specific incidence rates for MSM is problematic because of missing data on the denominator (the total number of MSM).[4, 5]

The systematic review and meta-analysis aims at systematically analysing data on the incidence of STIs during the follow-up of participants in published PrEP trials and cohort studies. These studies implement STI testing at regular intervals, often quarterly. The goal of this systematic review is to generate effect estimates on the incidence rates of different STIs for MSM who are eligible for PrEP because of engaging in risky sexual practices such as condomless anal intercourse with casual partners.

2. Search for studies

2.1. Databases and search dates

- Databases: MEDLINE (via OVID), EMBASE (via OVID), Cochrane CENTRAL
- Search dates: From inception to the date of the search

2.2. Search strategies

<u>MEDLINE</u>

1. preexposure prophylaxis.ab,ti,kw.
2. preexposure chemoprophylaxis.ab,ti,kw.
3. pre-exposure prophylaxis.ab,ti,kw.
4. pre-exposure chemoprophylaxis.ab,ti,kw.
5. PrEP.ab,ti,kw.
6. Pre-Exposure Prophylaxis/
7. 1 or 2 or 3 or 4 or 5 or 6
8. men who have sex with men.ab,ti,kw.
9. (men adj3 who have sex with men).ab,ti,kw.
10. MSM.ab,ti,kw.
11. Gay.ab,ti,kw.
12. Homosexuals.ab,ti,kw.
13. Homosexuality, Male/
14. Sexual Minorities/
15. anal intercourse.ab,ti,kw.

16. Unsafe Sex/
17. 8 or 9 or 10 or 11 or 12 or 13 or 14 or 15 or 16
18. 7 and 17

EMBASE

1. preexposure prophylaxis.ab,ti,kw.
2. preexposure chemoprophylaxis.ab,ti,kw.
3. pre-exposure prophylaxis.ab,ti,kw.
4. pre-exposure chemoprophylaxis.ab,ti,kw.
5. PreP.ab,ti,kw.
6. Pre-Exposure Prophylaxis/
7. 1 or 2 or 3 or 4 or 5 or 6
8. men who have sex with men.ab,ti,kw.
9. (men adj3 who have sex with men).ab,ti,kw.
10. MSM.ab,ti,kw.
11. Gay.ab,ti,kw.
12. Homosexuals.ab,ti,kw.
13. exp male homosexual/
14. exp men who have sex with men/
15. exp sexual minority/
16. anal intercourse.ab,ti,kw.
17. exp unsafe sex/
18. 8 or 9 or 10 or 11 or 12 or 13 or 14 or 15 or 16 or 17
19. 7 and 18

Cochrane CENTRAL

#1 preexposure prophylaxis:ti,ab,kw (Word variations have been searched)
#2 preexposure chemoprophylaxis:ti,ab,kw (Word variations have been searched)
#3 pre-exposure prophylaxis:ti,ab,kw (Word variations have been searched)
#4 pre-exposure chemoprophylaxis:ti,ab,kw (Word variations have been searched)
#5 PrEP:ti,ab,kw (Word variations have been searched)
#6 MeSH descriptor: [Pre-Exposure Prophylaxis] explode all trees
#7 #1 or #2 or #3 or #4 or #5 or #6
#8 men who have sex with men:ti,ab,kw (Word variations have been searched)
#9 men near/3 who have sex with men:ti,ab,kw (Word variations have been searched)
#10 MSM:ti,ab,kw (Word variations have been searched)
#11 Gay:ti,ab,kw (Word variations have been searched)
#12 Homosexuals:ti,ab,kw (Word variations have been searched)
#13 MeSH descriptor: [Homosexuality, Male] explode all trees
#14 MeSH descriptor: [Sexual Minorities] explode all trees
#15 anal intercourse:ti,ab,kw (Word variations have been searched)
#16 MeSH descriptor: [Unsafe Sex] explode all trees
#17 #8 or #9 or #10 or #11 or #12 or #13 or #14 or #15 or #16
#18 #7 and #17

3. Eligibility criteria for the inclusion of studies

- Longitudinal clinical studies (RCTs, clinically controlled studies, cohort studies) of PrEP in MSM at risk for the acquisition of HIV
- At least 3 months of follow-up reported
- Incident cases of STIs (not HIV) reported, as based on testing for STIs
- Sufficient data to calculate the number of person-years of follow-up
- Publication types: Full text or conference abstracts
- Publication languages: English, German, French
- Publication date: not limited
- Geographic location of studies: not limited
- Exclusion: data relying on self-reported incidence of STIs
- Exclusion: data only presented graphically

4. Methods for study selection and data collection

Identified records will be checked for eligibility using a two-step process, screening titles and abstracts first, and assessing full texts of records included thereafter. Data will be extracted independently by two evaluators. Disagreement will be resolved by discussion or by involving a third investigator. Trial reports as well as the study protocols (if available) will be considered. Data items:

- Type of study (participant-blinded placebo-controlled RCT / open-label placebo controlled RCT / active-controlled RCT / cohort study)
- Inclusion / exclusion criteria for participants in each study
- Geographic location
- Recruitment period
- Sample size
- Length of follow-up
- Counselling for sexual behaviour
- Assessment methods for the detection of STIs during the follow-up
- Baseline data of the sample (gender, sexual identity, age, race / ethnic group, education / income, alcohol and drug consumption, sexual behaviour, STI prevalence at baseline)
- Number of incident STI diagnoses during follow-up (any STI, at least one STI, syphilis, gonorrhoea, chlamydia, mycoplasma and ureaplasma infections (site-specific as reported), viral hepatitis A, B, and C, anogenital warts, genital herpes, other STI as reported in the studies
- Corresponding person-years of follow up

As data from a randomised controlled trial suggests that PrEP could have an effect on the incidence of genital herpes,[6] data on genital herpes will only be considered if reported separately for placebo or non-intervention arms. For data on hepatitis B, the same approach will be applied since tenofovir is an established medication for chronic hepatitis B.

5. Assessment of the quality of the data

In order to evaluate the quality of the data on STI incidence, three quality criteria will be applied: First, we will evaluate whether the data on incidence rates or numbers of incident STI diagnoses and person-years of follow-up are explicitly reported or easy to calculate or interpret. Second, we will critically appraise the methods used for the detection of STI during the follow-up ('application of robust methods for the detection of STI'). The evaluation will be based upon the Center for Disease Control (CDC) recommendations as derived from the '2015 Sexually Transmitted Diseases Treatment Guidelines' and will be assessed separately for each STI reported within the study. To satisfy this quality criterion, studies have to explicitly state having performed the following testing procedures at a minimum of six monthly intervals:

1. Syphilis serology for the detection of syphilis had to include a treponemal test (e.g. Treponema pallidum particle agglutination, TPPA or fluorescent treponemal antibody absorbed, FTA-ABS) or the combination of a nontreponemal test (e.g. rapid plasma reagin, RPR or Venereal Disease Research Laboratory, VDRL) for the detection and a treponemal test for the verification of the diagnosis.
2. Screening for gonorrhoea, chlamydia, mycoplasma, and ureaplasma had to include testing of the urine or a urethral swab, and rectal and pharyngeal swabs using nucleic acid amplification testing (NAAT).
3. Screening for viral hepatitis had to be based on serologic testing for hepatitis A and hepatitis C, and hepatitis B virus surface antigen to test for acute Hepatitis B virus infection.
4. Screening for anogenital warts, genital herpes, and other sexually transmitted infections had to include at least clinical inspection of the predilection sites. Data on genital warts and genital herpes will only be considered if studies explicitly report incident cases. Where this was not the case, we calculated mean prevalence rates instead of incidence rates.

Third, the study size will be evaluated, considering a study size of more than 500 person-years of follow-up as an indicator of high quality.

6. Calculation of incidence rates from studies

Incidence rates will be calculated as events per 100 person-years of follow-up. Assuming a Poisson distribution, we will use log incidence rates and corresponding standard errors to determine incidence rates with 95%-CIs. If no incident cases occurred during follow-up, we will add a correction of 0.5 to the number of cases and person-years of follow-up as described in similar studies.[7, 8]

If incidence rates with 95% confidence intervals (95%-CIs) and the total person-years of follow-up are not reported in the studies, person-years will be calculated as follows: If possible, we will multiply the number of participants per visit by the length of time since the previous visit, or we will multiply the number of participants by the mean follow-up time. Where only the overall number of participants at baseline and at the end of follow-up are reported and the number of participants for each time point is missing, we will calculate person-years by multiplying the number of participants who completed the study plus half the

number of participants who had not completed the closing visit by the length of the follow-up period.

7. Synthesis of the results from each study

STI incidence data will be grouped according to study type and geographic location. We will calculate pooled estimates of the incidence rates and 95% CIs using a random effects model (DerSimonian-Laird) to account for methodological and clinical differences between the studies. Heterogeneity will be quantified by means of the Higgins's I-squared test statistic.[9]

8. Heterogeneity and sensitivity analyses

To examine sources of heterogeneity, we will separately evaluate subgroups along study design and geographic location. A sensitivity analysis will be performed for each outcome, only considering data extracted from studies that explicitly report incidence rates and that report having applied STI screening methods that meet our quality criterion #2.

9. References

1. Gama A, Martins MO, Dias S. HIV Research with Men who Have Sex with Men (MSM): Advantages and Challenges of Different Methods for Most Appropriately Targeting a Key Population. *Aims Public Health* 2017;**4**:221-39.
2. Magnani R, Sabin K, Saidel T, Heckathorn D. Review of sampling hard-to-reach and hidden populations for HIV surveillance. *Aids* 2005;**19**:S67-S72.
3. Zhang D, Bi P, Hiller JE, Lv F. Web-based HIV/AIDS behavioral surveillance among men who have sex with men: Potential and challenges. *International Journal of Infectious Diseases* 2008;**12**:126-31.
4. Marcus U, Hickson F, Weatherburn P, Schmidt AJ. Estimating the size of the MSM populations for 38 European countries by calculating the survey-surveillance discrepancies (SSD) between self-reported new HIV diagnoses from the European MSM internet survey (EMIS) and surveillance-reported HIV diagnoses among MSM in 2009. *BMC Public Health* 2013;**13**:919.
5. Marcus U, Schmidt AJ, Kollan C, Hamouda O. The denominator problem: estimating MSM-specific incidence of sexually transmitted infections and prevalence of HIV using population sizes of MSM derived from Internet surveys. *BMC Public Health* 2009;**9**:181.
6. Celum C, Morrow RA, Donnell D, et al. Daily oral tenofovir and emtricitabine-tenofovir preexposure prophylaxis reduces herpes simplex virus type 2 acquisition among heterosexual HIV-1-uninfected men and women: a subgroup analysis of a randomized trial. *Annals of Internal Medicine* 2014;**161**:11-9.
7. Yousef F, Cardwell C, Cantwell MM, Galway K, Johnston BT, Murray L. The incidence of esophageal cancer and high-grade dysplasia in Barrett's esophagus: a systematic review and meta-analysis. *Am J Epidemiol* 2008;**168**:237-49.
8. Pesonen JS, Cartwright R, Mangera A, et al. Incidence and Remission of Nocturia: A Systematic Review and Meta-analysis. *Eur Urol* 2016;**70**:372-81.
9. Higgins JP, Thompson SG, Deeks JJ, Altman DG. Measuring inconsistency in meta-analyses. *Bmj* 2003;**327**:557-60.
