# Supplementary material for: Incidence of sexually transmitted infections in men who have sex with men and who are at substantial risk of HIV infection – A meta-analysis of data from trials and observational studies of HIV pre-exposure prophylaxis
Source: PLoS One. 2018 Dec 3;13(12):e0208107. doi: 10.1371/journal.pone.0208107 (PMC6277101; doi:10.1371/journal.pone.0208107)
Supplement: S2 File — Results on the outcomes ‘Participants diagnosed with at least one STI’, anogenital warts, herpes simplex and other STIs. (PDF) [file pone.0208107.s002.pdf]

Titel: Incidence rates of sexually transmitted infections in men who have sex with men and who are at substantial risk of HIV infection – a meta-analysis of data from trials and observational studies of HIV pre-exposure prophylaxis

Authors: Ricardo N. Werner[1], Matthew Gaskins[1], Alexander Nast[1], Corinna Dressler[1]

[1] Charité – Universitätsmedizin Berlin, corporate member of Freie Universität Berlin, Humboldt-Universität zu Berlin, and Berlin Institute of Health, Department of Dermatology, Venerology und Allergology, Division of Evidence based Medicine (dEBM)

S2 File: Online supplement

1. Participants diagnosed with at least one STI 2
2. Data on anogenitals warts, herpes simplex, and other STIs 2
3. References 4

1. Participants diagnosed with at least one STI

For the outcome '**participants diagnosed with at least one STI**', we obtained data from one placebo-controlled trial[1], the PrEP group of an open-label trial,[2] and four cohort studies.[3-6] This yielded a cumulative 2454.2 person-years of follow-up. Incidence rates ranged from 34.3/100py (95%-CI: 29.2-40.3) in the placebo-controlled RCT to 65.8/100py (95%-CI: 59.0-73.5) in a cohort study. Our overall pooled estimate was 52.5/100py (95%-CI: 41.9-65.8, I^2=93.6%). Data from the three studies conducted in Europe[2, 5, 6] had similar incidence rates with a pooled estimate of 63.3/100py (95%-CI: 58.5-68.4, I^2=0), whereas the incidence rates in two cohort studies conducted in the USA[3, 4] differed substantially: 40.2/100py (95%-CI: 36.2-44.7) and 63.0/100py (95%-CI: 52.4-75.7). When considering only the three studies that fulfilled the quality criteria for our sensitivity analysis,[2-4] we estimated incidence rates ranging from 40.2/100py (95%-CI: 36.2-44.7) to 63.0/100py (95%-CI: 52.3-75.7), with an overall pooled estimate of 53.8/100py (95%-CI: 38.8-74.6, I^2=93.2%).

2. Data on anogenitals warts, herpes simplex, and other STIs

Anogenital warts

One placebo-controlled RCT[7] reported data on condylomata acuminata, but whether only incident or all prevalent cases were counted was not specified. We used this study to calculate a mean point prevalence rate, which was 4.44% (95%-CI: 3.91-5.03). One study reported data on "anogenital HPV"[8]. Since this was not further specified, we did not include this data.

Herpes simplex

No study reported on the incidence of clinically manifest HSV-infection. Data for the incidence of HSV-2 seroconversion of participants who were seronegative at baseline were reported separately for the placebo group in one RCT[9]. During a follow-up of 1071py, a seroconversion rate of 5.6/100py (95%-CI: 4.4-7.2) was seen. One RCT reported the incidence of genital herpes without specification of the group allocation of the participant[8]; this data was therefore

not included. One study reported on the incidence of genital ulcers[7]. Since this outcome was not further specified and may imply several differential diagnoses, these data were not further evaluated.

Other STI

One placebo-controlled RCT[8] reported the incidence of one case of **molluscum contagiosum**. It remained unclear, whether this was a chance finding or whether participants were routinely checked for skin changes corresponding to molluscum contangiosum. One cohort study[10] reported that no cases of **trichomonas** occurred during follow-up. It was unclear, whether participants were routinely screened for trichomonas infection.

3. References

1. Molina JM, Capitant C, Spire B, et al. On-demand preexposure prophylaxis in men at high risk for HIV-1 infection. *New England Journal of Medicine* 2015;**373**:2237-46.
2. McCormack S, Dunn DT, Desai M, et al. Pre-exposure prophylaxis to prevent the acquisition of HIV-1 infection (PROUD): Effectiveness results from the pilot phase of a pragmatic open-label randomised trial. *The Lancet* 2016;**387**:53-60.
3. Golub SA, Pena S, Boonrai K, Douglas N, Hunt M, Radix A. STI data from community-based prep implementation suggest changes to CDC guidelines. *Topics in Antiviral Medicine* 2016;**24 (E-1)**:368.
4. Marcus JL, Hurley LB, Hare CB, et al. Preexposure Prophylaxis for HIV Prevention in a Large Integrated Health Care System: Adherence, Renal Safety, and Discontinuation. *J Acquir Immune Defic Syndr* 2016;**73**:540-6.
5. Molina JM, Charreau I, Spire B, et al. Efficacy, safety, and effect on sexual behaviour of on-demand pre-exposure prophylaxis for HIV in men who have sex with men: An observational cohort study. *The Lancet HIV* 2017.
6. Noret M, Balavoine S, Noret M, et al. Daily or on demand oral TDF/FTC for HIV pre-exposure prophylaxis: experience from an hospital-based clinic in France. *AIDS (London, England)* 2018.
7. Grant RM, Lama JR, Anderson PL, et al. Preexposure chemoprophylaxis for HIV prevention in men who have sex with men. *New England Journal of Medicine* 2010;**363**:2587-99.
8. Hosek S, Siberry G, Bell M, Lally M, Kapogiannis B, Green K. The acceptability and feasibility of an HIV pre-exposure prophylaxis (PrEP) trial with young men who have sex with men (YMSM). Journal of Acquired Immune Deficiency Syndromes. 2013; 62(4):[447-56 pp.]. Available from: http://onlinelibrary.wiley.com/o/cochrane/clcentral/articles/241/CN-00861241/frame.html.
9. Marcus JL, Glidden DV, McMahan V, et al. Daily oral emtricitabine/tenofovir preexposure prophylaxis and herpes simplex virus type 2 among men who have sex with men. *PLoS One* 2014;**9 (3)**.
10. Lalley-Chareczko L, Clark D, Conyngham SC, et al. Delivery of TDF/FTC for Pre-Exposure Prophylaxis to Prevent HIV-1 Acquisition in Young Adult Men Who Have Sex with Men and Transgender Women of Color using a Urine Adherence Assay. *Journal of Acquired Immune Deficiency Syndromes (1999)* 2018.
